# Effects of Fe^2+^ addition to sugarcane molasses on poly-γ-glutamic acid production in *Bacillus licheniformis CGMCC NO. 23967*

**DOI:** 10.1186/s12934-023-02042-0

**Published:** 2023-02-24

**Authors:** Lifei Guo, Liang Lu, Huichao Wang, Xiaoxing Zhang, Genan Wang, Tingbin Zhao, Guobao Zheng, Changsheng Qiao

**Affiliations:** 1 College of Bioengineering, Tianjin University of Science and Technology, Tianjin, 300457 China; 2 Tianjin Engineering Research Center of Microbial Metabolism and Fermentation Process Control, College of Biotechnology, Tianjin University of Science and Technology, Tianjin, 300457 China; 3 State Key Laboratory of Biobased Fiber Manufacturing Technology, Tianjin University of Science and Technology, Tianjin, 300457 People’s Republic of China; 4 Institute of Forestry Sciences Agricultural Biotechnology Research Center, Ningxia Academy of Agriculture and Forestry Science, Yinchuan, 750002 China; 5 Tianjin Huizhi Biotrans Bioengineering Co., Ltd, Tianjin, 300457 China

**Keywords:** Poly-γ-glutamic acid (γ-PGA), *Bacillus licheniformis*, Fermentation, *S*ugarcane molasses, FeSO_4_·7H_2_O, Metabolomics, Transcriptome

## Abstract

**Background:**

Poly-γ-glutamic acid (γ-PGA) is biodegradable, water-soluble, environment-friendly, and edible. Consequently, it has a variety of industrial applications. It is crucial to control production cost and increase output for industrial production γ-PGA.

**Results:**

Here γ-PGA production from sugarcane molasses by *Bacillus licheniformis CGMCC NO. 23967* was studied in shake-flasks and bioreactors, the results indicate that the yield of γ-PGA could reach 40.668 g/L in a 5L stirred tank fermenter. Further study found that γ-PGA production reached 70.436 g/L, γ-PGA production and cell growth increased by 73.20% and 55.44%, respectively, after FeSO_4_·7H_2_O was added. Therefore, we investigated the metabolomic and transcriptomic changes following FeSO_4_·7H_2_O addition. This addition resulted in increased abundance of intracellular metabolites, including amino acids, organic acids, and key TCA cycle intermediates, as well as upregulation of the glycolysis pathway and TCA cycle.

**Conclusions:**

These results compare favorably with those obtained from glucose and other forms of biomass feedstock, confirming that sugarcane molasses can be used as an economical substrate without any pretreatment. The addition of FeSO_4_·7H_2_O to sugarcane molasses may increase the efficiency of γ-PGA production in intracellular.

**Supplementary Information:**

The online version contains supplementary material available at 10.1186/s12934-023-02042-0.

## Background

Poly-γ-glutamic acid (γ-PGA) is a natural polymer composed of l-glutamic acid, d-glutamic acid, or both monomers in different ratios and repetition frequency. The molecular weight of γ-PGA generally ranges from 10 to 1000 kDa [1]. This polymer is thought to be environmentally friendly, and shows good biocompatibility, ion adsorption, and biodegradability [2]. It is used for thickening, gel and film formation, moisturizing, and adhesion, and is most often used in cosmetics, food processing, agriculture, medicine, environmental protection and other fields [3–6]. γ-PGA was first discovered in the capsule of *Bacillus anthracis* by Ivanovics and Bruckner [7]. Subsequently, γ-PGA has also been found in the fermentation extracts of *B. subtilis* and *B. nattobacter* as well [8]. γ-PGA is mainly prepared via chemical synthesis [9], enzyme extraction [10], enzyme conversion [11], and microbial fermentation [12]. Microbial fermentation is low-cost, highly efficient, and highly scalable, and has therefore become the main method of γ-PGA preparation at present [13].

Low-cost fermentation substrates and high-yielding strains are the basis of large-scale industrial production. These methods produce γ-PGA using low-cost substrates such as rice [14], sago [15], and corn cobs [16, 17]. However, starch and raw lignocellulosic materials must first be heated and enzymatically treated to release fermentable sugars, which increases the processing cost. The operons responsible for γ-PGA synthesis were first identified in *Bacillus subtilis* and include pgsB -C -A (or -AA) and -E genes. PgsB has been shown to hydrolyze ATP to ADP in the presence of L-glutamate; PgsC is the membrane embedded component of the γ-PGA synthesis system. PgsA has a membrane-anchored region and a region corresponding to the position occupied by divalent cations in serine/threonine phosphatase, with which it shows substantial sequence similarity. Therefore, the accumulation of γ-PGA in the medium may change in the presence of metal ions (including Mg^2+^ Zn^2+^, and Mn^2+^). Zn^2+^ can increase the production of γ-PGA by enhancing the expression of PgsE, which is an important part of the γ-PGA synthesis pathway [18]. According to a series of preliminary experiments showed that other metal ions (K^+^, Ca^2+^, Mg^2+^, etc.) had NO effect on γ-PGA fermentation of *Bacillus licheniformis CGMCC NO. 23967*, but only Fe^2+^ had a significant effect (Additional file 1).

In this study, we aimed to explore the effect of sugarcane molasses and exogenous Fe^2+^ treatment on γ-PGA production by *B. licheniformis*, and to develop an economical and environmentally friendly bioprocess for γ-PGA production that used sugarcane molasses as feedstock. Sugarcane molasses is a byproduct of the sugar industry, and is mainly composed of sucrose, along with some glucose and fructose. It can be directly used for fermentation without pretreatment [19, 20]. In this paper, we report the fermentation kinetics of *B. licheniformis* when sugarcane molasses is used as the main carbon source. We then used metabolomic and transcriptomic analyses to gain insight into the mechanism at work and to analyze the change in the key intracellular metabolites of *B. licheniformis* after the addition of Fe^2+^. The results will help to improve the efficiency and specificity of γ-PGA production.

## Results and discussion

### γ-PGA fermentation in shake-flasks

**Fig. 1:**
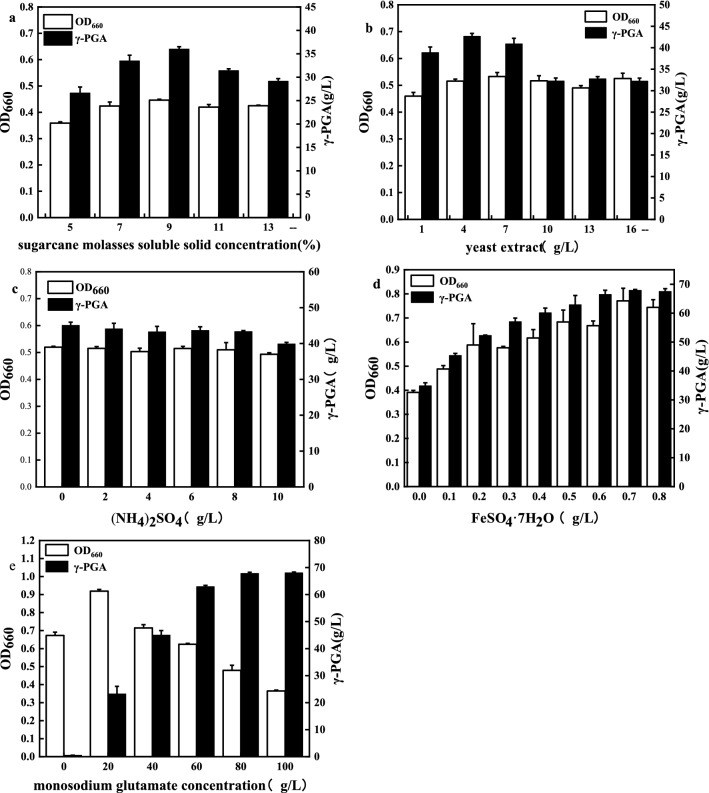
γ-PGA production from diluted sugarcane molasses in shake-flasks. **a** Effect of sugarcane molasses soluble solid concentration, **b** Effect of nitrogen source (yeast extract), **c** Effect of nitrogen source ((NH_4_)_2_SO_4_, **d** Effect of FeSO_4_·7H_2_O concentration, **e** Effect of monosodium glutamate concentration. Data reported were averages from triplicate run were averages from triplicate run

Figure 1 shows the effect of different concentrations of sugarcane molasses (5–13% soluble solids), nitrogen supplementation (1–16 g/L yeast extract, 0–10 g/L (NH_4_)_2_SO_4_), FeSO_4_·7H_2_O concentration (0–0.8 g/L), and monosodium glutamate concentration (0–100 g/L) on γ-PGA production in shake-flask fermentations.

### Effect of sugarcane molasses concentration

When the concentration of sugarcane molasses soluble solids increased from 5 to 9%, we found that γ-PGA yield continuously increases, from 26.632 to 36.022 g/L, and stimulates cell growth (Fig. 1a). However, further increasing the concentration of sugarcane molasses soluble solids to 13% led to a decrease in γ-PGA yield and biomass. This suggests that either sugarcane molasses soluble solids themselves (since they may cause osmotic stress) or other inhibitors present in sugarcane molasses extracts cause inhibition of the fermentation reaction [21].

### Effect of nitrogen supplementation

Different γ-PGA-producing bacteria utilize nitrogen sources differently. For example, *B. myloliquefaciens LL3* [22] and *B. subtilis TAM-4* [23] can produce γ-PGA from inorganic nitrogen sources. Moreover, *Bacillus subtilis CGMCC 0833* [24] and *Bacillus sp. RKY3* [25] can use organic nitrogen sources to produce γ-PGA. In this study, adding (NH_4_)_2_SO_4_ had no effect on the γ-PGA yield (Fig. 1c), but adding 4 g/L yeast extract to the sugarcane molasses substrate gave the highest γ-PGA production (42.697 g/L), and the yeast extract maintained favorable cell growth conditions (Fig. 1b). The addition of more than 4/L yeast extract resulted in a decrease in γ-PGA production; this indicated that the sugarcane molasses substrate contained sufficient nitrogen to support cell growth, but only a small amount of nitrogen source was needed to support γ-PGA production.

### The effect of exogenous FeSO_4_·7H_2_O supplementation

Figure 1d shows the effect of the addition of FeSO_4_·7H_2_O on γ-PGA production. At a concentration of FeSO_4_·7H_2_O ranging from 0 to 0.7 g/L, the production of γ-PGA increased with increasing FeSO_4_·7H_2_O, from 34.886 to 67.891 g/L. However, when the concentration of FeSO_4_·7H_2_O was higher than 0.7 g/L, there were no obvious changes in γ-PGA yield, and therefore we concluded that the concentration of FeSO_4_·7H_2_O no longer had a significant effect on γ-PGA yield. A concentration of 0.7 g/L was thus selected as the optimal concentration of FeSO_4_·7H_2_O. Because the addition of FeSO_4_·7H_2_O to sugarcane molasses had a great effect on γ-PGA yield, we studied the effect of adding FeSO_4_·7H_2_O to the 5L bioreactor on γ-PGA synthesis.

### The effect of monosodium glutamate concentration

γ-PGA synthesis strains can be divided into glutamate-dependent [26] and non-glutamate-dependent [23] strains. The latter does not require exogenous addition of glutamate; for these strains, the cost of the growth medium is lower, but its γ-PGA yield is also lower. Therefore, current production research focuses mainly on glutamate-dependent strains. In this study, *B. licheniformis CGMCC3967* was used as a representative glutamate-dependent strain. At low concentration, monosodium glutamate (MSG) had a significant effect on γ-PGA yield, when the concentration of MSG was higher than 80 g/L, there were no obvious changes in γ-PGA yield. Therefore, from the economic point of view, selected 80 g/L MSG for fermentation (Fig. 1e).

### γ-PGA fermentation in a bioreactor

In shake-flask studies, we determined that γ-PGA fermentation with diluted sugarcane molasses (9% soluble solids) without pretreatment with nitrogen supplementation (4 g/L yeast extract), 0.7 g/L FeSO_4_·7H_2_O, and 80 g/L monosodium glutamate, were the optimal conditions.

### The effect of FeSO_4_·7H_2_O concentration

Since we observed that the addition of FeSO_4_·7H_2_O to the sugarcane molasses substrate had a great effect on γ-PGA yield, we studied the effect of adding FeSO_4_·7H_2_O to the 5L bioreactor on γ-PGA synthesis.

**Fig. 2:**
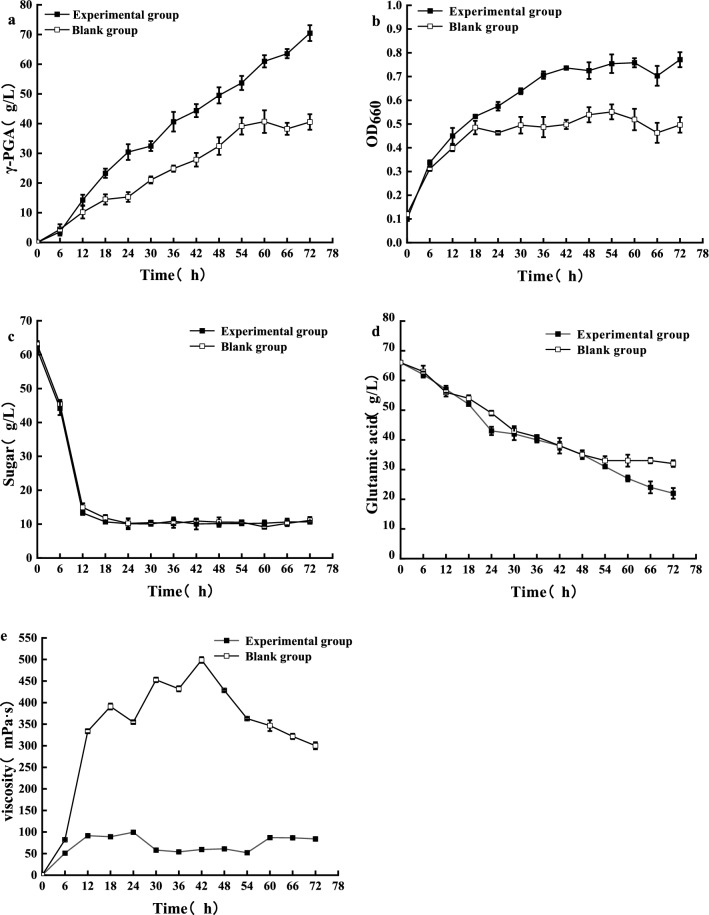
The effect of **a** γ-PGA production, **b** biomass, consumption of **c** sugar and **d** glutamic acid, **e** viscosity adding FeSO_4_·7H_2_O to 5L bioreactor

The effect of FeSO_4_·7H_2_O on γ-PGA fermentation was studied in a 5L fermenter (Fig. 2). We compared a blank to an experimental group, and the optimal medium determined in the shake-flask was used as the experimental group. The blank group had the same composition as the experimental group with one critical difference: FeSO_4_·7H_2_O was not added. For the blank group, γ-PGA production reached 40.668 g/L within 72 h, with a productivity of 0.565 g/L·h. For the experimental group, the γ-PGA production reached 70.436 g/L within 72 h, with a productivity of 0.979 g/L·h (Fig. 2a). γ-PGA was no longer synthesized in the blank group after 60 h, while γ-PGA was synthesized at a high rate in the experimental group until the end of fermentation. At the end of fermentation, γ-PGA production in the experimental group had increased by 73.8% compared with the blank group. Moreover, the experimental group also showed higher biomass (Fig. 2b) and lower residual glutamate (Fig. 2d) than the blank group, indicating that adding FeSO_4_·7H_2_O facilitates higher γ-PGA production. The viscosity of the fermentation broth decreased after adding FeSO_4_·7H_2_O (Fig. 2e), which may be related to the molecular weight of γ-PGA. If the fermentation liquid is too thick, oxygen is dissolved poorly, which results in insufficient oxygen supply and ultimately affects fermentation [27]. Based on our 5L fermenter results, we found that the addition of FeSO_4_·7H_2_O to the sugarcane molasses substrate significantly affected biomass, fermentation broth viscosity, glutamate consumption, and γ-PGA yield, and we followed this experiment with metabolic and transcriptomic analyses.

### Effect of aeration and agitation

Agitation and aeration are important for all aerobic processes, and have a significant effect on the yield of most biopolymers [28, 29]. Both agitation and aeration are involved in overall mass and oxygen transfer in the process fluid to different extents. Agitation controls nutrient transfer and the distribution of air and oxygen, while aeration determines the oxygenation of the culture and also contributes to bulk mixing of the fermentation fluid [30].

**Fig. 3:**
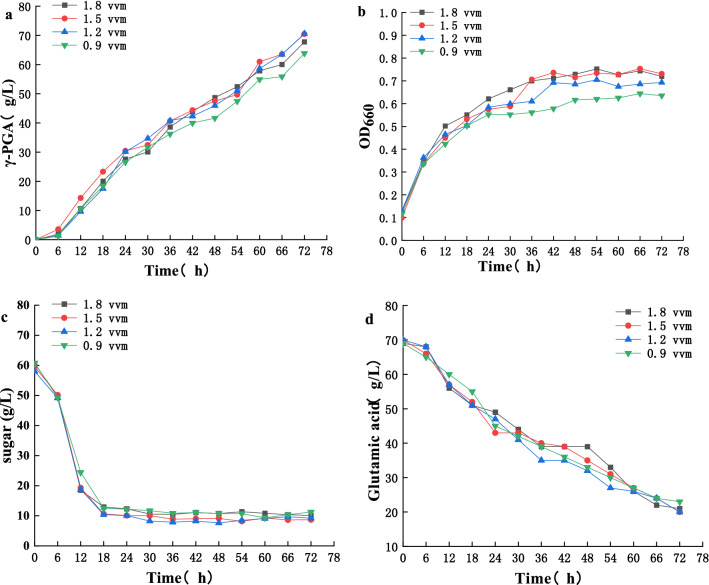
The effect of **a** γ-PGA production, **b** biomass, consumption of **c** sugar and **d** glutamic acid in batch cultures of *B. licheniformis CGMCC NO. 23967* under different aeration conditions

**Fig. 4:**
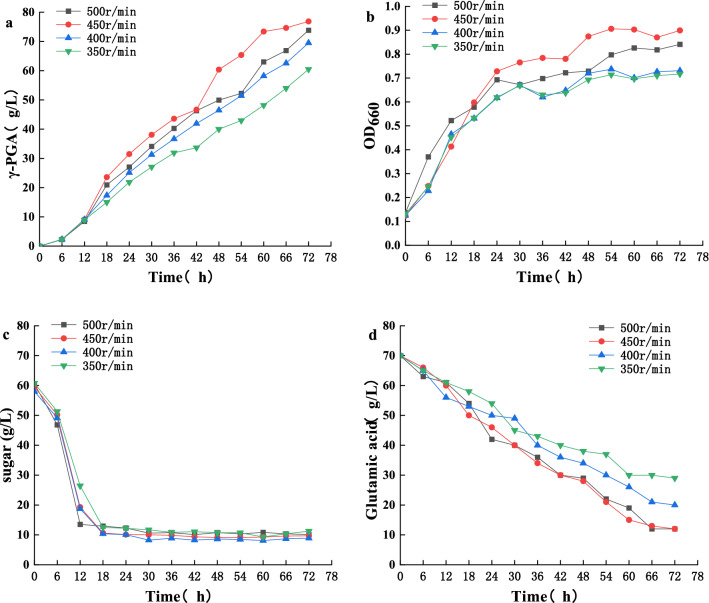
The effect of **a** γ-PGA production, **b** biomass, consumption of **c** sugar and **d** glutamic acid in batch cultures of *B. licheniformis CGMCC NO. 23967* under different agitation conditions

We then studied the trends of γ-PGA yield, biomass, total sugar, and residual glutamate under different aeration (Fig. 3) and agitation conditions (Fig. 4). At a base speed of 400 rpm and aeration from 0.9 to 1.2 vvm, γ-PGA yield (Fig. 3a), biomass (Fig. 3b), sugar (Fig. 3c), and the sodium glutamate utilization rate (Fig. 3d) were all increased. With continuous improvement in aeration, we observed increased biomass but decreased γ-PGA yield. After the optimal aeration of 1.2 vvm was obtained, we studied the stirring speed, and found that PGA yield (Fig. 4a), biomass (Fig. 4b), sugar (Fig. 4c), and sodium glutamate utilization rate (Fig. 4d) increased from 350 to 450 rpm. Thus, maximum γ-PGA production of 76.848 g/L and productivity of 1.07 g/L·h were obtained at an agitation speed of 450 rpm and an aeration rate of 1.2 vvm. These results revealed that *B. licheniformis CGMCC NO. 23967* growth was closely linked to the aeration rate and agitation speed. Under higher aeration and agitation conditions, *B. licheniformis CGMCC NO. 23967* uses nutrients for growth rather than γ-PGA production [30]. In this study, the maximum γ-PGA produced (76.848 g/L) was much greater than the L-glutamate consumed (58 g/L), indicating that *B. licheniformis CGMCC NO. 23967* produced some glutamate itself. Compared with other strategies for γ-PGA production (Table 1), Fe^2+^ addition to sugarcane molasses in this study provide the γ-PGA production is the highest and more economical.

**Table 1:** Comparison of γ-PGA production and productivity among different strains

Strain	Main nutrients	Fermentation process	Production (g/L)	Productivity (g/L/h)	References
*B. subtilis* 2756	l-glutamic acid, sago, yeast extract	Flasks	39.8	0.415	[15]
*B. subtilis* NX-2	Glucose, l-glutamic acid, yeast extract,	Fed-batch fermentation (7.5L fermentor)	73.0	0.81	[14]
*B. subtilis* SCP010–1	Corncob hydrolysate, monosodium glutamate, yeast extract	Fed-batch fermentation (2L flask)	30.035	0.601	[16]
*B. subtilis* 242	Cane molasses, l-glutamic, corn steep liquor	Fed-batch fermentation (5L fermentor)	32.14	0.67	[20]
*B. siamensis* IR10	l-glutamic acid, molasses, NH_4_Cl	Fed‑batch and repeated fed‑batch fermentation (3L fermentor))	41.4–45.42	1.67–2.02	[19]
*B. siamensis* SB1001	Sucrose, l-glutamic acid	250 ml flasks	25.22	1.05	[31]
*B. licheniformis* WX-02	Glycerol, sodium glutamate, NaNO_3_, citric acid	Fed-batch fermentation (5L fermentor)	36.83	0.767	[26]
*B. licheniformis* CGMCC NO. 23967	Monosodium glutamate, sugarcane molasses, yeast extract	Batch fermentation (5L fermentor)	76.848	1.07	This work

### Metabolomic analyses of *B. licheniformis* cultured with sugarcane molasses supplemented with FeSO_4_·7H_2_O

#### Intracellular metabolites of *B. licheniformis* were detected by GC–MS

First, the intracellular metabolites of thalli at different time points were analyzed. Samples were taken at three fermentation time points: 24 h, 42 h, and 66 h. The thalli of samples from the experimental and blank groups were examined using GC–MS. A total of more than 150 peaks were detected, and 39 kinds of intracellular metabolites were accurately matched with signatures from the NIST 11 database. These metabolites included amino acids (n = 12) fatty acids (n = 5), organic acids (n = 11), sugars (n = 6), as well as nucleic acids and other compounds (Table 2).

**Table 2:** Names and classifications of intracellular metabolites of *Bacillus licheniformis* detected

Classification	Name of metabolite	
Amino acids	Alanine	Glycine
	Ornithine	Valine
	Serine	Threonine
	Proline	Leucine
	Glutamic	Aspartic acid
	Arginine	Lysine
Fatty acids	Palmitic acid	Pentanoic acid
	Stearic acid	Heptadecanoic acid
	Hexadecanoic acid	
Organic acids	Propionic acid	Succinic acid
	Fumaric acid	Pyruvate
	Oxalic acid	Gluconic acid
	Aconitic acid	Lactic acid
	Acetic acid	2-Aminocaprylic acid
	4-Aminobutanoic acid	
Saccharides	Galactose	Fructose
	Glucose	Lactose
	Lyxose	Mannitol
Others	Inositol	Urea
	Adenosine	Butane
	Butylamine	

### PCA and PLS-DA were used to identify differences in intracellular metabolite profiles before and after adding FeSO_4_·7H_2_O

To compare the effects of FeSO_4_·7H_2_O on the in vivo metabolism of *B. licheniformis*, we selected three parallel samples at 24 h, 42 h, and 66 h to generate intracellular metabolite profiles. GC–MS data were normalized by MetaboAnalyst 5.0 Statistical Analysis, and subsequently unsupervised principal component analysis (PCA) and supervised partial least square discriminant analysis (PLS-DA) were used to analyze differences in metabolite profiles at different time points.

VIP analysis was carried out along with the PLS-DA model, and the differential metabolites were sorted according by contribution rate. Those metabolites with a general variable VIP value greater than 1 were deemed to be significant biomarkers. After 24 h of fermentation, the metabolites with different contribution rates (from high to low) were: acetic acid, lactic acid, glutamic acid, ornithine, palmitic acid, 4-aminobutyric acid, proline, inositol, succinic acid, oxalic acid, mannitol, fructose, lysine, stearic acid, and glycine. After 42 h of fermentation, the metabolites with different contribution rates (again from high to low) were: acetic acid, stearic acid, 4-aminobutyric acid, mannitol, lysine, adenosine, fructose, valine, glutamic acid, leucine, palmitic acid, succinic acid, 2-aminobutyric acid, glycine, and serine. Finally, after 66 h of fermentation, the metabolites with different contribution rates were: glutamic acid, mannitol, stearic acid, 4-aminobutyric acid, palmitic acid, lysine, leucine, lactic acid, guanosine, proline, adenosine, succinic acid, oxalic acid, inositol, and 2-aminocaprylic acid.

**Fig. 5:**
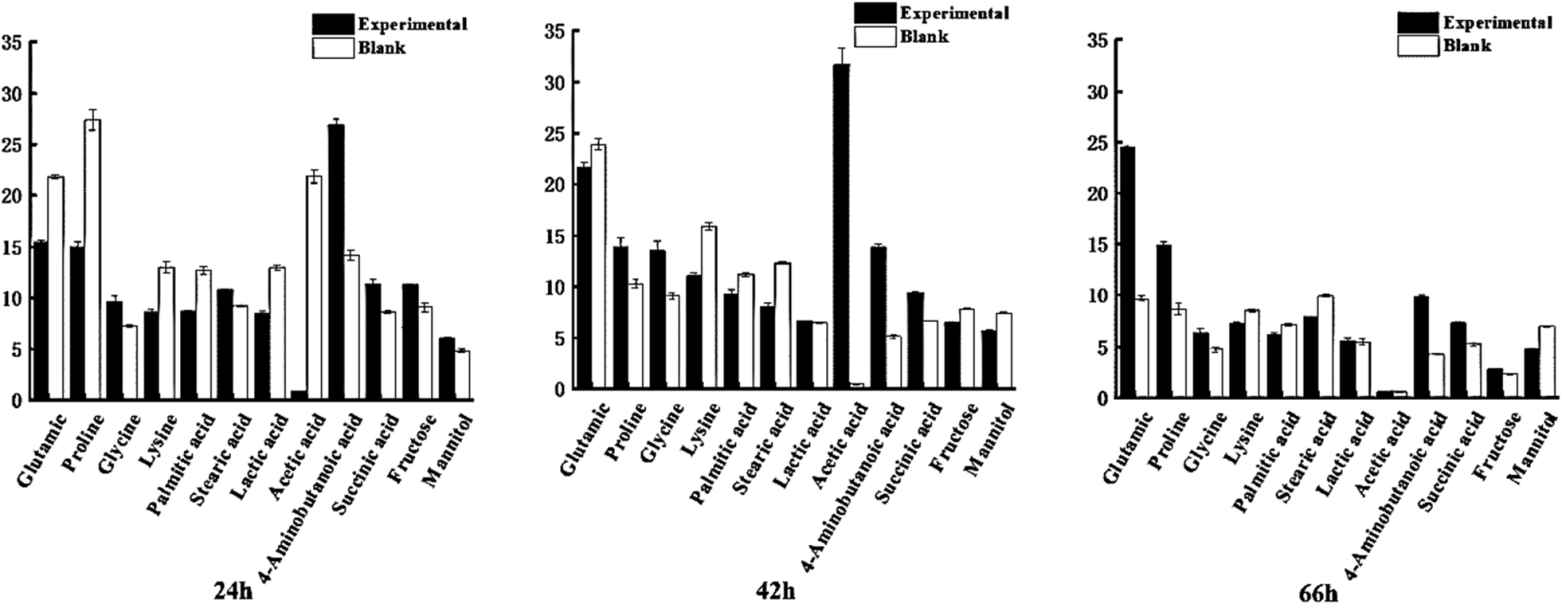
Comparison of relative concentrations of differential metabolites at different time points

Glycolysis, the pentose phosphate pathway (PPP), the tricarboxylic acid (TCA) cycle, amino acid metabolism, and glutamate synthesis are all involved in the biosynthesis of γ-PGA [32].γ-PGA is a secondary metabolite, and any increase in the metabolic rate of γ-PGA producing cells is likely to increase its yield [33]. Therefore, we then analyzed the relative contents of different metabolites related to *B. licheniformis* in sugarcane molasses with or without FeSO_4_·7H_2_O (i.e., the experimental and blank groups; Fig. 5). In this study, the metabolic pathways associated with differential metabolites in the experimental and blank group at different time points mainly included γ-PGA synthesis, glycolysis, the tricarboxylic acid cycle, and fatty acid metabolism.

### Changes in metabolites related to amino acids

Comparing the blank and experimental groups, different amino acid metabolites identified at different time points included glutamic acid, proline, glycine, and lysine. Glutamate is a direct precursor of γ-PGA synthesis, and its intracellular content and metabolism directly affect γ-PGA synthesis [34]. When glutamic acid is absent from the medium, it is mainly produced by α-ketoglutaric acid in the TCA cycle [35]. Before 42 h of fermentation, the relative glutamate content of the experimental and blank groups did not differ, but both increased with longer fermentation time. When the fermentation time reached 66 h, the glutamate content of the experimental group continued to increase with longer fermentation time, while the glutamate content of the blank group decreased significantly (Fig. 5). Combined with the fermentation results from the 5L fermenter (Fig. 2), we conclude that FeSO_4_·7H_2_O stimulates intracellular glutamate synthesis, thereby increasing γ-PGA synthesis. Oxygen supply is the bottleneck of γ-PGA-producing bacterial cultures. Thus, as the concentration of dissolved γ-PGA increases, the viscosity of the culture medium increases, so the generation of γ-PGA gradually makes the transfer of oxygen from the aerated gas to the culture medium more difficult [33]. Proline metabolism plays an important role in maintaining intracellular reactive oxygen species (ROS) balance [36–38]. In addition, proline can be converted to glutamate by proline dehydrogenase. After 42 h of fermentation, the experimental group maintained a higher relative proline content than the blank group (Fig. 5). Higher proline content can ensure subsequent glutamate reserve, maintain the balance of intracellular ROS, and therefore ensure the continuity of γ-PGA synthesis. After 66 h of fermentation, the relative content of both glutamate and proline in the experimental group remained high, while both glutamate and proline content of the blank group decreased sharply (Fig. 5). Combined with observed changes of γ-PGA production in the 5L fermenter (Fig. 2a), these results suggest that FeSO_4_·7H_2_O stimulated the synthesis of glutamate and proline in *B. licheniformis* cells, thereby enhancing γ-PGA synthesis. γ-PGA synthesis requires energy, and therefore requires ATP [33]. Glycine can be converted into pyruvate through a transaminase, and can then enter the pyruvate or TCA cycles. At different time points, the relative glycine content of the experimental group was higher than the blank group (Fig. 5). This indicates that FeSO_4_·7H_2_O stimulated intracellular glycine synthesis, thereby enhancing the TCA cycle. The intracellular lysine content of the experimental group was lower than that of the blank group at different time points (Fig. 5), indicating that the flux from α -ketoglutarate to lysine was decreased and the flux from α-ketoglutarate to glutamate was increased in the experimental group, thus increasing the production of γ-PGA.

### Metabolite changes in glycolysis pathway

The glycolysis pathway is critical to carbon consumption and to growth rates. Therefore it further affects γ-PGA productivity [39]. Both fructose and mannitol are related to glycolysis pathway, and the intracellular content of both decreased as fermentation time increased (Fig. 5). After 42 h of fermentation, the fructose and mannitol content of the blank group increased, suggesting that there was not enough ATP to catalyze fructose conversion in the blank group, which evidently led to the accumulation of intermediate glycolysis products. In the glycolysis cycle, if oxygen is insufficient, acetic acid is produced. After 24 h, acetic acid content in the blank group increased significantly. This indicates that under the influence of viscosity generated by the fermentation process, the amount of dissolved oxygen became low, which reduced the rate of increase of bacterial volume. As fermentation continued, dissolved oxygen was stable in the fermentation broth and acetic acid was consumed by glycolysis. In the experimental group, this process occurred at 42 h, at which time the volume of bacteria in the experimental group reached a stable stage, indicating that the growth period of bacteria was prolonged, and the growth and metabolic activity of the bacteria were enhanced.

### TCA cycle metabolite changes

The TCA cycle produces large amounts of ATP and other intermediates required for other anabolic pathways (e.g., amino acid biosynthesis) [40]. Succinic acid is an important metabolite in the TCA cycle. The relative content of succinic acid in the experimental group was higher than in the blank group at different time points (Fig. 5). This indicated that the TCA cycle of the experimental group was very active. In other words, the addition of FeSO_4_·7H_2_O to sugarcane molasses promoted the TCA cycle in *B. licheniformis* and enhanced growth, metabolic activity, and γ-PGA synthesis.

### Fatty acid related metabolites changes

An increase in medium chain fatty acids and a decrease in long chain fatty acids can improve the fluidity of the cell membrane, and greater membrane fluidity is conducive to the secretion of γ-PGA [41]. The stearic acid and palmitic acid content of the experimental group was found to be reduced (Fig. 5), which facilitates the secretion of γ-PGA into extracellular spaces and relieves feedback inhibition. Fatty acid synthesis requires large quantities of acetyl-CoA and ATP. The fact that stearic acid and palmitic acid content decreased in the experimental group indicates that more acetyl-CoA flowed into the TCA cycle to provide energy for γ-PGA synthesis and thallus growth.

### Changes in other intracellular metabolites

4-Aminobutyric acid is a precursor of glutamic acid in the glutamic acid metabolic pathway. 4-aminobutyric acid can be converted to glutamic acid by glutamic acid decarboxylase, or it can be converted to succinate by 4-aminobutyrate transaminase and succinate hemi-aldehyde dehydrogenase, whereafter it can enter the TCA cycle. The 4-aminobutyric acid content of the experimental group was higher than the blank group at different time points (Fig. 5). This suggests that the addition of FeSO_4_·7H_2_O to the sugarcane molasses substrate increased 4-aminobutyric acid synthesis, guaranteeing the supply of glutamic acid and succinic acid, and thereby promoting the synthesis of γ-PGA.

### Transcriptomic analyses of γ-PGA synthesis after the addition of FeSO_4_·7H_2_O to a sugarcane molasses substrate

#### Sequencing data quality assessment

Samples from the blank and experimental groups were sequenced using an Illumina NovaSeq 6000 high-throughput sequencer. The quality assessment of sample sequencing output data is shown in Table 3. The obtained base quality values Q30 and Q20 were both high, and the error rates of sample sequencing results were lower than 0.03%, which satisfy quality requirements for Illumina sequencing.

**Table 3:** Transcriptome sequencing data quality

Name	Clean reads	Clean base(G)	Error rate (%)	Q20 (%)	Q30 (%)
Blank.1	15,335,530	4.6	0.03	97.59	93.01
Blank.2	6,573,445	1.97	0.03	97.54	93.14
Blank.3	14,660,138	4.4	0.03	97.62	93.03
FeSO_4_·7H_2_O.1	11,282,889	3.38	0.03	97.76	93.40
FeSO_4_·7H_2_O.2	17,460,739	5.24	0.03	97.52	92.85
FeSO_4_·7H_2_O.3	14,126,731	4.24	0.03	97.62	93.13

Clean Base: Total number of bases; Q20 Q30: The percentage of bases with Phred greater than 20 and 30 in the total number of bases

### Differential gene expression analysis

The differentially expressed gene dataset was statistically analyzed. Differentially expressed genes (DEGs) with multiples of difference > 1.5 and P-values < 0.05 were identified. These included 108 up-regulated genes and 114 down-regulated genes. The identification of this many DEGs indicated that adding FeSO_4_·7H_2_O to the culture medium had a significant effect on gene expression in the thallus.

### GO analysis

**Fig. 6:**
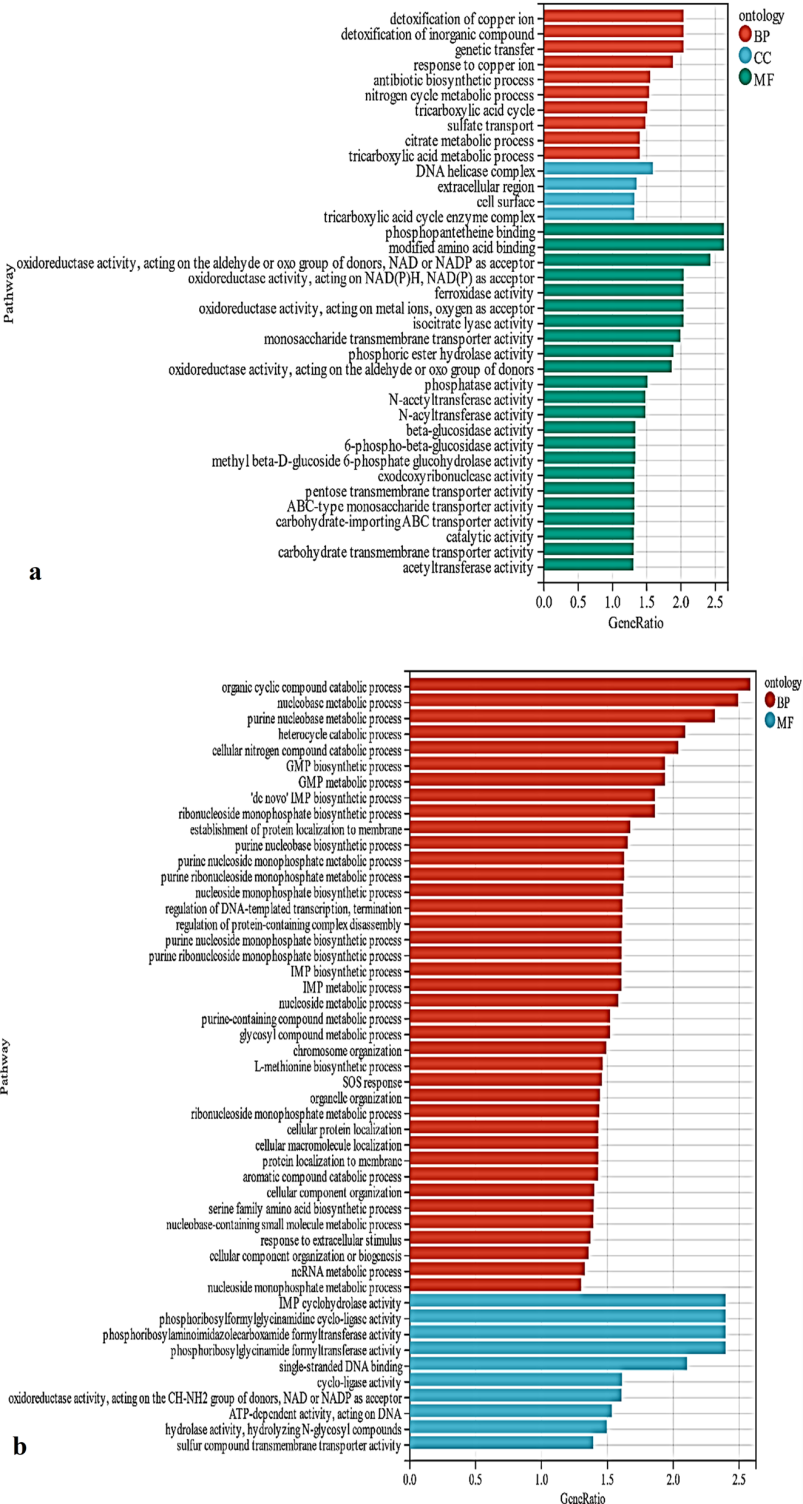
**a** GO enrichment analysis of up-regulated expression of differential genes, **b** GO enrichment analysis of down-regulated expression of differential genes

Next, we used the expression levels of protein-coding genes in different samples to perform GO enrichment analysis on the sample data from the experimental and blank group. DEGs from these groups were classified as belonging to the molecular function (MF), biological process (BP), or cell component (CC) GO categories. The abscissa represents the degree of enrichment of differential genes (− log10 (P-value)), and the ordinate represents the functional annotation of the DEG (i.e., BP, CC, or MF; Fig. 6a, b).

**Fig. 7:**
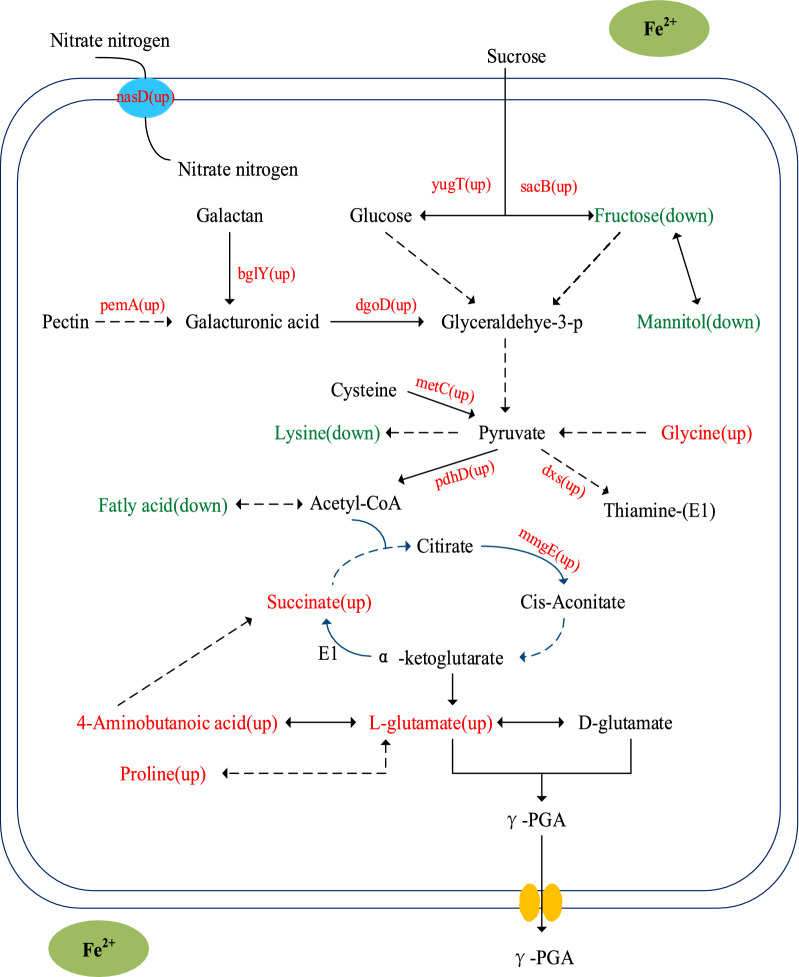
Effect of FeSO_4_·7H_2_O on the metabolic pathway and gene expression of *B.licheniformis* (→ Represents further reaction, ⇢Stands for multistep reaction, Red is up; Green is down, E1 stands for α-ketoglutarate dehydrogenase)

As shown in Fig. 7, when FeSO_4_·7H_2_O was added, the up-regulated DEGs were mainly involved in the TCA cycle, glucose metabolism, transmembrane transport of substances, and organic acid metabolism. The cell components (CC) categories associated with these DEGs were cell surface and tricarboxylic acid cyclase complex, while the molecular function categories associated with the upregulated DEGs included reductase, transferase, and transporter activities. The down-regulated DEGs were found to be mainly involved in purine metabolism, protein localization, organic cyclic catabolism, and IMP metabolism. The molecular functions of the enriched DEGs mainly included ligase activity, oxidoreductase activity, and hydrolase activity.

Next, we combined this DEG dataset with KEGG pathway analysis, and the analyzed the fold differences of the DEGs identified. Table 4 shows the DEGs involved in the major metabolic pathways involved in γ-PGA synthesis.

**Table 4:** Statistics of differential genes

Gene	Gene function	Gene expression
bglY	Beta-galactosidase BglY	Up
odhA	2-Oxoglutarate dehydrogenase E1 component	Up
pemA	Pectin esterase A	Up
dxs	1-Deoxy-d-xylulose-5-phosphate synthase	Up
nasD	Nitrate transport protein NasD	Up
mmgE	Citrate/2-methylcitrate dehydratase	Up
gpml	2,3-Bisphosphoglycerate-independent phosphoglycerate mutase	Up
yugT	Oligo-1,6-glucosidase 3	Up
metC	Cystathionine beta-lyase Met C	Up
sacB	Levansucrase	Up
dgoD	d-Galactonate dehydratase	Up
pdhD	Dihydrolipoyl dehydrogenase	Up

Sucrose (the main carbon source found in molasses) can be hydrolyzed to glucose and fructose by α-glucosidase and l-sucrase. Moreover, fructose can be interconverted with glucose by xylose isomerase, and β-galactosidase catalyzes the hydrolysis of galactose to other forms of galactose. In this pathway, glyceraldehyde 3-phosphate is formed from galactodehydrogenase and galactodehydrase, where it can then enter the glycolysis pathway. Furthermore, glucose can be phosphorylated into glucose 6-phosphate via hexokinase, whereupon it can also enter the glycolysis pathway to provide energy for cells. Finally, 2, 3-diphosphoglycerate independent phosphoglycerate mutase can catalyze 3-phosphoglycerate to 2-phosphoglycerate. We found that the gene expression levels of α-glucosidase, l-sucrase, galactose dehydrogenase and 2, 3-diphosphoglycerate independent phosphoglycerate transmutase were all up-regulated, and the glycolysis metabolic pathway was accelerated (Fig. 7). In the experimental group, the intracellular fructose metabolite consumption was faster, and more carbon was directed to cell growth and γ-PGA synthesis.

Pectinase can hydrolyze the pectin polymer in sugarcane molasses into pectin monomers. These monomers can then be converted to galacturonic acid via a multi-step reaction, which can then in turn be catalyzed by galactose dehydrase into glyceraldehyde 3-phosphate and enter the glycolysis pathway (Fig. 7). After FeSO_4_·7H_2_O supplementation (experimental group), we found that pectinase gene expression was upregulated. Therefore, the available carbon sources of *B. licheniformis* were increased, and the growth and metabolism of *B. licheniformis* were accelerated. Both of these changes were conducive to increased synthesis of γ-PGA products.

Pyruvate, as a precursor of acetyl-CoA, is very important for the TCA cycle. In addition to pyruvate production by glycolysis, cysteine β -lyase can also be cleaved to pyruvate. pdhD is a gene involved in energy conversion that is often upregulated to promote cell survival [42]. In addition, pdhD expression in the presence of dihydroacyl dehydrogenase can promote the conversion of pyruvate to acetyl-coA, which is conducive to the synthesis of citric acid. Citrate dehydrogenase (2-methylcitrate dehydrogenase) catalyzes the formation of cis-aconite acid from citric acid. 2-oxogglutarate dehydrogenase (α-ketoglutarate dehydrogenase) promotes the conversion of α -ketoglutarate to succinyl-coA, thereby increasing the flux from 2-oxogglutarate to glutamate [43]. In the experimental group, we found that the expression of the genes coding for cysteine β-lyase, dihydroacyl dehydrogenase, citrate/2-methylcitrate dehydrase, and 2-oxyglutarate dehydrogenase were all up-regulated (Table 4). The result was to enhance the intensity of the metabolic activity associated with the TCA cycle *in B. licheniformis*, which was beneficial to cell growth and γ-PGA synthesis.

Thiamine is a very important cofactor, and is directly linked to the activity of key growth and metabolism enzymes such as α-ketoglutarate dehydrogenase. 1-deoxy-d-xylose-5-phosphosynthase can promote the synthesis of thiamine from pyruvate and glyceraldehyde 3-phosphate. The increased expression of the gene coding for 1-deoxy-d-xylose-5-phosphate synthase indicated that the TCA cycle and glutamic acid synthesis were enhanced in the experimental group, which was also beneficial to γ-PGA synthesis.

Nitrate can be used as a nitrogen source for cell growth and intracellular glutamate synthesis [26]. In addition, it can be employed as an electron acceptor during ATP generation, especially under oxygen-limiting conditions [44, 45]. The addition of nitrate to glucose-based media can promote extracellular glutamate assimilation and intracellular glutamate synthesis. Moreover, it can also enhance energy supply, thereby improving γ-PGA biosynthesis [46]. In this study, the expression of nitrate transporter genes was associated with increased cellular nitrogen sources and promoted both cell growth and the synthesis of γ-PGA.

In conclusion, when FeSO_4_·7H_2_O was added to the fermentation medium of the experimental group, the expression levels of many genes were affected (Fig. 7). Taken together, our results suggest that the total effect of these changes was to enhance biological metabolism and γ-PGA product synthesis (Additional file 1).

### Association analysis between metabolomic and transcriptomic data

The effect of FeSO_4_·7H_2_O on the synthesis of γ-PGA by *Bacillus licheniformis CGMCC NO. 23967* was analyzed by metabolomics and transcriptomics. We then analyzed the relationship between the two omics datasets generated by these analyses.

The decrease in fructose and mannitol detected by our metabolomic analysis indicates that fructose consumption increased and more carbon sources entered the glycolysis pathway in the experimental group. At the same time, our transcriptomic dataset found up-regulated gene expression for the genes coding for α-glucosidase, l-sucrase, galactose dehydrase, and 2,3-diphosphoglycerate independent phosphoglycerate translocation enzyme, which also accelerated the metabolic activity associated with the glycolysis pathway. In the experimental group, our metabolomic analysis also found increased glycine, succinic acid, and 4-aminobutyric acid levels, but decreased lysine levels. Taken together, this shows that more pyruvate flowed to the TCA cycle, which enhanced the TCA cycle, and thereby provided more energy for cell growth and metabolism. At the same time, our transcriptomic results found that the expression of the genes coding for cythionine β-lyase, dihydroacyl dehydrogenase, citrate/2-methylcitrate dehydrase, and 2-oxyglutarate dehydrogenase were all up-regulated. We also found that increased TCA cycle metabolic intensity was beneficial to cell growth and metabolism and γ-PGA product synthesis.

In the experimental group, our metabolomic analyses detected increased glutamic acid content, γ-PGA synthesis of direct precursors increased, and an overall significant increase in the amount of γ-PGA synthesized. At the same time, our transcriptomic analyses detected the up-regulation of 2-oxogglutarate dehydrogenase gene expression, resulting in increased flux from 2-oxogglutarate to glutamate. In summary, the conclusions of our metabolomic and transcriptomic analyses agree with and can verify each other. Taken together as a whole, our results indicate that the addition of FeSO_4_·7H_2_O to the medium enhanced the glycolysis pathway and TCA cycle, promoted cell growth, increased intracellular glutamic acid content, and provided sufficient energy and precursor metabolites for γ-PGA synthesis.

## Conclusions

γ-PGA can be produced by *B. licheniformis* from diluted sugarcane molasses without any pretreatment. The results indicate that γ-PGA production reached 40.668 g/L in a 5L stirred tank fermenter. Further study found that γ-PGA production reached 70.436 g/L after FeSO_4_·7H_2_O was added. The highest γ-PGA production observed was 76.848 g/L, and a productivity of 1.07 g/L·h can be attained at 1.2 vvm and 450 rpm, which offers an economical process for industrial production of bio-based γ-PGA. The conversion of metabolites related to the TCA cycle and the glycolysis pathway were enhanced after the addition of FeSO_4_·7H_2_O. Moreover, we identified 12 genes related to γ-PGA biosynthesis were found to be up-regulated. Therefore, we speculated that the addition of FeSO_4_·7H_2_O to sugarcane molasses is an effective stimulatory agent for cell growth and γ-PGA biosynthesis in *B. licheniformis*.

## Methods

### Microorganisms and media used

*Bacillus licheniformis CGMCC3967* was isolated in our laboratory, then preserved in the China General Microbiological Culture Collection Center (Beijing, China; sample No. CGMCC3967). The strain was stored in potato dextrose agar (PDA) slants at 4 °C and was subcultured every 2 weeks. The seed medium contained (in g/L): glucose 30, yeast extract 7, K_2_HPO_4_·3H_2_O 0.5, and MgSO_4_·7H_2_O 0.5. The pH was adjusted to 7.2–7.3 by 6 M NaOH, after which the media was sterilized by autoclaving at 121 °C for 20 min. The glucose solution was sterilized separately at 115 °C for 20 min, then added to the sterile medium before inoculation. The seed culture was cultivated in a 500 ml shake-flask containing 100 ml medium and was incubated in a rotary shaker at 37 °C, 220 rpm for 15 h. The fermentation medium contained (in g/L): monosodium glutamate 70, glucose 30, yeast extract 7, peptone 5, (NH_4_)_2_SO_4_ 7, K_2_SO_4_ 15, CaSO_4_·2H_2_O 0.5, MgSO_4_·7H_2_O 0.5, FeSO_4_·7H_2_O 0.1, and glycerin at a concentration of 60 mL/L. This medium was adjusted to pH 7.2–7.3 before being sterilized at 121 °C for 20 min. Finally, a monosodium glutamate solution was sterilized separately at 115 °C for 20 min and was then added to the prepared sterile medium before inoculation.

### Sugarcane molasses as a fermentation substrate

In this study we used sugarcane molasses as a fermentation substrate. This molasses contained approximately 56% total sugar, 7.45% reducing sugar, 3.33% total nitrogen, 0.85% ammonia nitrogen, 4.45% K^+^, 0.63% Ca^+^, 0.47% Mg^2+^, 0.083% Fe^2+^ and 1.35% amino acids. Molasses could only be used as the main fermentation substrate after dilution with water to adjust the soluble solids content. After this dilution, sugarcane molasses was used as a fermentation substrate for both shake-flasks (500 ml) or stirred-tank bioreactors.

### Fermentation kinetics studies

Fermentation kinetics was first studied in shake-flasks. For these experiments, the sugarcane molasses ranged from 5 to 13% soluble solids, and nitrogen was supplemented at varying quantities (1–16 g/L yeast extract, 0–10 g/L (NH_4_)_2_SO_4_), along with monosodium glutamate concentration (0–100 g/L), and FeSO_4_·7H_2_O concentration (0–0.8 g/L). Each flask containing 50 ml medium was inoculated with a 15 h seed culture at 10% (v/v). After inoculation, flasks were incubated at 37 °C, 220 rpm for 72 h. Three flasks were used for each condition studied to create independent technical replicates.

We then investigated γ-PGA fermentation kinetics in a 5L stirred tank fermenter (GRJB-5D, Zhenjiang Gree Co., Ltd., China) using diluted sugarcane molasses (9% soluble solids) without pretreatment, since this was determined as the optimal condition by the previous shake-flask experiment. For these experiments the fermenter contained 3L fermentation medium inoculated with 300 mL seed culture, and the reaction was controlled at 37 °C with agitation at 400 rpm and aeration at 1.5vvm. The effect of aeration and agitation on γ-PGA production was evaluated by varying the aeration rate from 0.9 ~ 1.8 vvm and the agitation speed from 350 to 500 rpm.

### Fermentation parameter assays and γ-PGA production

Biomass was determined by optical density measurement at 660 nm, and the fermentation broth was diluted 25 times with deionized water before measurement. The viscosity of the fermentation liquid was measured using a viscometer (NDJ-8S). Soluble solid content was measured using a hand-held sugar meter, and total sugar was determined by the phenol–sulfuric acid method [47].

Next, the cells were separated by centrifugation, and after 100-fold dilution, the concentration of the remaining glucose and l-glutamate in the fermentation broth were analyzed using a SBA-40E biosensor equipped with glucose and l-glutamate oxidase electrodes.

During fermentation, broth (5 mL) was collected at different time points and centrifuged at 15,000 rpm. The resulting supernatant (1 mL) was diluted 50 times. These sample were then analyzed by high performance liquid chromatography (HPLC; L-2000, Hitachi Ltd., Japan). HPLC was performed using a SB-800 HQ gel permeation chromatography column at 30 °C eluted with 50 mM Na_2_SO_4_ at a rate of 0.5 mL/min. Detection was at 210 nm and the purified γ-PGA was used as a standard. All liquids entering the HPLC column were filtered using a 0.22um membrane.

### Extraction of intracellular metabolites and metabolomic analysis

We obtained blank and experimental group fermentation broth samples (50 mL) from the 5 L stirred tank fermenter after 24, 44, and 66 h of fermentation to examine intracellular metabolites. Samples were then centrifuged at 15,000 rpm for 20 min. Next, the precipitate was collected, 4 mL PBS buffer was used to re-suspend the thalli, and centrifugation was repeated 2–3 times. The collected precipitate was then ground in liquid nitrogen for 25 min, after which the cellular fragment (150 mg) of the resulting grind was collected and mixed with 1 mL of precooled 60% methanol. This mixture was then centrifuged at 12,000 rpm at − 4 °C for 10 min. After derivatization, the mixture was subjected to refrigerated centrifugation at 10,000 rpm at − 4 °C for 5 min, and the resulting supernatant was prepared for gas chromatography–mass spectrometry (GC–MS) analysis after storage for 2 h at 25 °C.

GC–MS is a widely used, highly efficient and sensitive method for the analysis of complex biological mixtures [48]. The original GC–MS data were imported into AMDIS software for deconvolution. The NIST8.0 database was searched to estimate the relative content of identifiable metabolites. Multivariate data analysis was then conducted after standardization by SPSS (version XYZ, IBM SPSS Inc. USA).

### Transcriptomic analysis

RNA extraction, library establishment and sequencing: fermentation broth samples were taken and centrifuged in 10 mL centrifuge tubes at 13,000 r/min for 30 min, after which the supernatant was removed and the thallium was suspended in normal saline at 13,000 r/min for 30 min. This process was repeated twice. Total RNA was then extracted from *Bacillus licheniformis* cells using a TRIzol kit, and the integrity and total amount of isolated RNA were measured using an Agilent 2100 BioAnalyzer (Agilent Technologies, CA, USA). mRNA was then purified by probe and randomly interrupted by divalent cations to construct a cDNA library. These libraries were then subjected to quality control analyses. Samples were then sequenced on an Illumina Nova Seq 6000 if they passed the quality inspection.

Sequencing data quality statistics and sequence comparisons: Fastp (version 0.19.7) was used to obtain basic statistics regarding the quality of the original reads, and Trinity 2.11.0 was used to conduct sequence comparisons.

Statistical analyses of data: DESeq2 software was used for statistical analysis of gene differences in the original dataset and to identify the differentially expressed genes (DEGs) between samples. The GO and KEGG databases were used to sort the differential genes, to obtain functional annotations of DEGs, and to analyze the relatedness between metabolic pathways.

## Supplementary Information

**Additional file 1.** Effect of K^+^, Ca^2+^ and Mg^2+^ concentration on γ-PGA fermentation.

## Data Availability

All data generated or analyzed during this study are included in this article.

## References

[1] Wang L, Chen S, Yu B (2022). Poly-γ-glutamic acid: recent achievements, diverse applications and future perspectives. Trends Food Sci Technol.

[2] Li M, Zhang Z, Li S, Tian Z, Ma X (2021). Study on the mechanism of production of gamma-PGA and nattokinase in *Bacillus subtilis* natto based on RNA-seq analysis. Microb Cell Fact.

[3] Yu J, Wu N, Zheng X, Zheng M (2020). Preparation of water-soluble chitosan/poly-gama-glutamic acid-tanshinone IIA encapsulation composite and its in vitro/in vivo drug release properties. Biomed Phys Eng Express.

[4] Yu Y, Yan F, Chen Y, Jin C, Guo JH, Chai Y (1811). Poly-gamma-glutamic acids contribute to biofilm formation and plant root colonization in selected environmental isolates of *Bacillus subtilis*. Front Microbiol.

[5] Kumar A, Mir SM, Aldulijan I, Mahajan A, Anwar A, Leon CH, Terracciano A, Zhao X, Su TL, Kalyon DM (2021). Load-bearing biodegradable PCL-PGA-beta TCP scaffolds for bone tissue regeneration. J Biomed Mater Res B Appl Biomater.

[6] Park S-B, Sung M-H, Uyama H, Han DK (2021). Poly(glutamic acid): Production, composites, and medical applications of the next-generation biopolymer. Prog Polym Sci.

[7] Parati M, Khalil I, Tchuenbou-Magaia F, Adamus G, Mendrek B, Hill R, Radecka I (2022). Building a circular economy around poly(D/L-gamma-glutamic acid)—a smart microbial biopolymer. Biotechnol Adv.

[8] Li D, Hou L, Gao Y, Tian Z, Fan B, Wang F, Li S (2022). Foods.

[9] Kumar R, Pal P (2015). Fermentative production of poly (gamma-glutamic acid) from renewable carbon source and downstream purification through a continuous membrane-integrated hybrid process. Bioresour Technol.

[10] Yao J, Xu H, Shi N, Cao X, Feng X, Li S, Ouyang P (2010). Analysis of carbon metabolism and improvement of gamma-polyglutamic acid production from *Bacillus subtilis* NX-2. Appl Biochem Biotechnol.

[11] Luo Z, Guo Y, Liu J, Qiu H, Zhao M, Zou W, Li S (2016). Microbial synthesis of poly-gamma-glutamic acid: current progress, challenges, and future perspectives. Biotechnol Biofuels.

[12] Ogunleye A, Bhat A, Irorere VU, Hill D, Williams C, Radecka I (2015). Poly-gamma-glutamic acid: production, properties and applications. Microbiology (Reading).

[13] Fang J, Liu Y, Huan C, Xu L, Ji G, Yan Z (2020). Comparison of poly-γ-glutamic acid production between sterilized and non-sterilized solid-state fermentation using agricultural waste as substrates. J Clean Prod.

[14] Tang B, Lei P, Xu Z, Jiang Y, Xu Z, Liang J, Feng X, Xu H (2015). Highly efficient rice straw utilization for poly-(gamma-glutamic acid) production by *Bacillus subtilis* NX-2. Bioresour Technol.

[15] Mohanraj R, Gnanamangai BM, Ramesh K, Priya P, Srisunmathi R, Poornima S, Ponmurugan P, Robinson JP (2019). Optimized production of gamma poly glutamic acid (γ-PGA) using sago. Biocatal Agric Biotechnol.

[16] Chang F, Li W, Hu H, Ge F, Chen G, Ren Y (2022). Chemical pretreatment and saccharification of corncob for poly-γ-glutamic acid production by *Bacillus subtilis* SCP010-1. Process Saf Environ Prot.

[17] Sun JD, Tang C, Zhou J, Wei P, Wang YJ, An W, Yan ZY, Yong XY (2021). Production of poly-gamma-glutamic acid (gamma-PGA) from xylose-glucose mixtures by *Bacillus **amyloliquefaciens* C1. 3 Biotech.

[18] Yamashiro D, Yoshioka M, Ashiuchi M (2011). Bacillus subtilis pgsE (Formerly ywtC) stimulates poly-gamma-glutamate production in the presence of zinc. Biotechnol Bioeng.

[19] Wang D, Kim H, Lee S, Kim DH, Joe MH (2020). High-level production of poly-gamma-glutamic acid from untreated molasses by *Bacillus siamensis* IR10. Microb Cell Fact.

[20] Li J, Chen S, Fu J, Xie J, Ju J, Yu B, Wang L (2022). Efficient molasses utilization for low-molecular-weight poly-gamma-glutamic acid production using a novel *Bacillus subtilis* stain. Microb Cell Fact.

[21] Xia J, Xu J, Hu L, Liu X (2016). Enhanced poly(L-malic acid) production from pretreated cane molasses by *Aureobasidium pullulans* in fed-batch fermentation. Prep Biochem Biotechnol.

[22] Cao M, Geng W, Liu L, Song C, Xie H, Guo W, Jin Y, Wang S (2011). Glutamic acid independent production of poly-gamma-glutamic acid by *Bacillus amyloliquefaciens* LL3 and cloning of pgsBCA genes. Bioresour Technol.

[23] Ito Y, Tanaka T, Ohmachi T, Asada Y (2014). Glutamic acid independent production of poly(γ-glutamic acid) by *Bacillus subtilis* TAM-4. Biosci Biotechnol Biochem.

[24] Wu Q, Xu H, Shi N, Yao J, Li S, Ouyang P (2008). Improvement of poly(gamma-glutamic acid) biosynthesis and redistribution of metabolic flux with the presence of different additives in *Bacillus subtilis* CGMCC 0833. Appl Microbiol Biotechnol.

[25] Jung DY, Jung S, Yun JS, Kim JN, Wee YJ, Jang HG, Ryu HW (2005). Influences of cultural medium component on the production of poly(γ-glutamic acid) by *Bacillus* sp. RKY3. Biotechnol Bioprocess Eng.

[26] Li X, Yang H, Zhou M, Zhan Y, Liu J, Yan D, Cai D, Chen S (2021). A novel strategy of feeding nitrate for cost-effective production of poly-γ-glutamic acid from crude glycerol by *Bacillus licheniformis* WX-02. Biochem Eng J.

[27] Sirisansaneeyakul S, Cao M, Kongklom N, Chuensangjun C, Shi Z, Chisti Y (2017). Microbial production of poly-gamma-glutamic acid. World J Microbiol Biotechnol.

[28] Cromwick AM, Birrer GA, Gross RA (1996). Effects of pH and aeration on gamma-poly(glutamic acid) formation by *Bacillus licheniformis* in controlled batch fermentor cultures. Biotechnol Bioeng.

[29] Huang W-C, Chen S-J, Chen T-L (2006). The role of dissolved oxygen and function of agitation in hyaluronic acid fermentation. Biochem Eng J.

[30] Bajaj IB, Singhal RS (2010). Effect of aeration and agitation on synthesis of poly (γ-glutamic acid) in batch cultures of *Bacillus licheniformis* NCIM 2324. Biotechnol Bioprocess Eng.

[31] Wang D, Hwang J-S, Kim D-H, Lee S, Kim D-H, Joe M-H (2020). A newly isolated *Bacillus siamensis* SB1001 for mass production of poly-γ-glutamic acid. Process Biochem.

[32] Sha Y, Sun T, Qiu Y, Zhu Y, Zhan Y, Zhang Y, Xu Z, Li S, Feng X, Xu H (2019). Investigation of glutamate dependence mechanism for poly-gamma-glutamic acid production in *Bacillus subtilis* on the basis of transcriptome analysis. J Agric Food Chem.

[33] Cao M, Feng J, Sirisansaneeyakul S, Song C, Chisti Y (2018). Genetic and metabolic engineering for microbial production of poly-gamma-glutamic acid. Biotechnol Adv.

[34] Zhang W, He Y, Gao W, Feng J, Cao M, Yang C, Song C, Wang S (2015). Deletion of genes involved in glutamate metabolism to improve poly-gamma-glutamic acid production in *B. **amyloliquefaciens* LL3. J Ind Microbiol Biotechnol.

[35] Commichau FM, Gunka K, Landmann JJ, Stulke J (2008). Glutamate metabolism in *Bacillus subtilis*: gene expression and enzyme activities evolved to avoid futile cycles and to allow rapid responses to perturbations of the system. J Bacteriol.

[36] Liang X, Zhang L, Natarajan SK, Becker DF (2013). Proline mechanisms of stress survival. Antioxid Redox Signal.

[37] Natarajan SK, Zhu W, Liang X, Zhang L, Demers AJ, Zimmerman MC, Simpson MA, Becker DF (2012). Proline dehydrogenase is essential for proline protection against hydrogen peroxide-induced cell death. Free Radic Biol Med.

[38] Zhang L, Alfano JR, Becker DF (2015). Proline metabolism increases katG expression and oxidative stress resistance in *Escherichia coli*. J Bacteriol.

[39] Mitsunaga H, Meissner L, Palmen T, Bamba T, Buchs J, Fukusaki E (2016). Metabolome analysis reveals the effect of carbon catabolite control on the poly(gamma-glutamic acid) biosynthesis of *Bacillus licheniformis* ATCC 9945. J Biosci Bioeng.

[40] Hada K, Hirota K, Inanobe A, Kako K, Miyata M, Araoi S, Matsumoto M, Ohta R, Arisawa M, Daitoku H (2019). Tricarboxylic acid cycle activity suppresses acetylation of mitochondrial proteins during early embryonic development in *Caenorhabditis elegans*. J Biol Chem.

[41] Li L, Liu Y, Jiang L, Ding S, Chen G, Liang Z, Zeng W (2021). Effects of cell physiological structure on the fermentation broth viscosity during poly-gamma-glutamic acid production by *Bacillus subtilis* GXA-28. Appl Biochem Biotechnol.

[42] Guo J, Cheng G, Gou XY, Xing F, Li S, Han YC, Wang L, Song JM, Shu CC, Chen SW, Chen LL (2015). Comprehensive transcriptome and improved genome annotation of *Bacillus licheniformis* WX-02. FEBS Lett.

[43] Hsueh YH, Huang KY, Kunene SC, Lee TY (2017). Poly-gamma-glutamic acid synthesis, gene regulation, phylogenetic relationships, and role in fermentation. Int J Mol Sci.

[44] Simon J, van Spanning RJ, Richardson DJ (2008). The organisation of proton motive and non-proton motive redox loops in prokaryotic respiratory systems. Biochim Biophys Acta.

[45] Berks BC, Page MD, Richardson DJ, Reilly A, Cavill A, Outen F, Ferguson SJ (1995). Sequence analysis of subunits of the membrane-bound nitrate reductase from a denitrifying bacterium: the integral membrane subunit provides a prototype for the dihaem electron-carrying arm of a redox loop. Mol Microbiol.

[46] Li X, Gou X, Long D, Ji Z, Hu L, Xu D, Liu J, Chen S (2014). Physiological and metabolic analysis of nitrate reduction on poly-gamma-glutamic acid synthesis in *Bacillus licheniformis* WX-02. Arch Microbiol.

[47] Dubois M, Gilles KA, Hamilton JK, Rebers PA, Smith F (1956). Colorimetric method for determination of sugars and related substances. Anal Chem.

[48] Papadimitropoulos MP, Vasilopoulou CG, Maga-Nteve C, Klapa MI (2018). Untargeted GC–MS metabolomics. Methods Mol Biol.

