# Supplementary material for: Effects of Fe2+ addition to sugarcane molasses on poly-γ-glutamic acid production in Bacillus licheniformis CGMCC NO. 23967
Source: Microb Cell Fact. 2023 Feb 24;22:37. doi: 10.1186/s12934-023-02042-0 (PMC9960700; doi:10.1186/s12934-023-02042-0)
Supplement: Supplementary file 1 — Additional file 1. Effect of K+, Ca2+ and Mg2+ concentration on γ-PGA fermentation. [file 12934_2023_2042_MOESM1_ESM.docx]

Effect of K_2_SO_4_concentration on γ-PGA fermentation

Effect of CaSO_4_·2H_2_O concentration on γ-PGA fermentation

Effect of MgSO_4_·7H_2_O concentration on γ-PGA fermentation
